# How individuals change during internet‐based interventions for depression: A randomized controlled trial comparing standardized and individualized feedback

**DOI:** 10.1002/brb3.1484

**Published:** 2019-11-27

**Authors:** Pavle Zagorscak, Manuel Heinrich, Johannes Bohn, Jana Stein, Christine Knaevelsrud

**Affiliations:** ^1^ Department of Clinical‐Psychological Intervention Freie Universität Berlin Berlin Germany

**Keywords:** depression, expectations, growth mixture modeling, Internet‐based interventions, patterns of change, social support

## Abstract

**Background:**

Standardized and individualized Internet‐based interventions (IBI) for depression yield significant symptom improvements. However, change patterns during standardized or individualized IBI are unknown. Identifying subgroups that experience different symptom courses during IBI and their characteristics is vital for improving response.

**Methods:**

Mildly to moderately depressed individuals according to self‐report (*N* = 1,089) were randomized to receive module‐wise feedback that was either standardized or individualized by a counselor within an otherwise identical cognitive‐behavioral IBI for depression (seven modules over six weeks). Depressive symptoms were assessed at baseline and before each module (Patient Health Questionnaire; PHQ‐9). Other individual characteristics (self‐report) and the presence of an affective disorder (structured clinical interview) were assessed at baseline. Growth mixture modeling was used to identify and compare subgroups with discernable change patterns and associated client variables across conditions.

**Results:**

Model comparisons suggest equal change patterns in both conditions. Across conditions, a group of immediate (62.5%) and a group of delayed improvers (37.5%) were identified. Immediate improvers decreased their PHQ‐9 score by 5.5 points from pre to post, with 33% of improvement occurring before treatment commenced. Delayed improvers were characterized by stable symptom severity during the first two modules and smaller overall symptom decrease (3.4 points). Higher treatment expectations, a current major depressive disorder (interview), and lower social support were associated with delayed improvement.

**Conclusion:**

Internet‐based interventions for depression with individualized and with standardized feedback lead to comparable patterns of change. Expectation management and bolstering of social support are promising strategies for individuals that are at risk for delayed improvement.

## Graphical abstract

The study investigates depressive symptom courses during IBI for depression with individualized versus standardized feedback. Across conditions, a group of immediate (62.5%) and a group of delayed improvers (37.5%) were identified. Higher treatment expectations, a current major depressive disorder and lower social support were associated with delayed improvement.

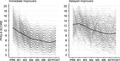

## 1 INTRODUCTION

The World Health Organization (2017) identified depression as the leading cause of disability worldwide. Even in high‐income countries, only one in five depressed individuals receives adequate treatment (Thornicroft et al., 2017). Different researchers (e.g., Kazdin, 2018) proposed Internet‐based interventions (IBI) as one approach to circumvent individual‐level barriers like problems with transportation, inconvenient treatment hours, and locations (i.e., long distance from home) and fear of stigma that impede the uptake of evidence‐based treatments (Harvey & Gumport, 2015). Meta‐analyses confirm the efficacy of standardized and individualized IBI for depression (e.g., Karyotaki et al., 2018, 2017). However, research on why, when, and how individuals improve throughout IBI with varying levels of individualization is lacking.

Identifying individuals who improve during IBI and their sociodemographic and clinical characteristics is a prerequisite for offering interventions that are tailored to the needs of specific populations and thus might increase response rates (Khan, Faucett, Lichtenberg, Kirsch, & Brown, 2012; Manen et al., 2015; Mueller et al., 2018). Moreover, learning about the particular point during treatment (and the associated intervention elements) at which certain individuals change is essential to advance the understanding of the underlying mechanisms of change (Klein & Kotov, 2016; Silberschatz, 2015). Growth mixture modeling (GMM) is a statistical approach that addresses these questions. It explores whether populations with heterogeneous symptom trajectories contain distinct homogenous subgroups (e.g., Jung & Wickrama, 2008; Muthén, 2006).

While GMM has been regularly used to investigate depressive symptom courses during face‐to‐face psychotherapy (e.g., Rubel, Lutz, & Schulte, 2015), there has only been a limited number of trials on this topic in IBI for depression (Batterham et al., 2018; Lutz et al., 2017; Sunderland, Wong, Hilvert‐Bruce, & Andrews, 2012). Sunderland et al. (2012) and Batterham et al. (2018) found two discernable symptom trajectories during IBI, with 75%–81% of individuals showing improvement and the remainder showing no or low symptom improvements. Divergently, Lutz et al. (2017) found three distinct groups of depressed individuals. One group improved immediately after baseline assessment (45%), another after being randomized to the intervention and registered on the website (39%), and a third showed early symptom deterioration (16%). The differing number of identified subgroups might be due to significant differences in study design and interventions under research. For instance, Lutz et al. (2017) focused on early symptom change before and during the first quarter of IBI. The authors modeled change from screening through registration and at week two and week four of treatment. Sunderland et al. (2012) and Batterham et al. (2018) aimed to explore the heterogeneity in symptom trajectories beyond the early stages of treatment. In addition, the studies differed with regard to the provided intervention. While Lutz et al. (2017) and Sunderland et al. (2012) focused on individuals with depression and anxiety, Batterham et al. (2018) treated depressive symptom load as secondary outcome in an intervention focusing on reducing suicidal thoughts. Another critical difference between the three studies pertains to the level of individualization offered. Batterham et al. (2018) and Sunderland et al. (2012) evaluated a self‐guided treatment (i.e., standardized, without regular guidance or feedback by clinicians) and Lutz et al. (2017) provided more severely depressed individuals with additional guidance (individualized weekly e‐mail support).

Since the intensity of guidance is considered to be one of the most central moderators of outcome in IBI for depression (e.g., Johansson & Andersson, 2012), more research is necessary to assess the influence of contact quantity and quality on patterns of change. Consequently, the current study investigates depressive symptom courses and their associations with pre‐interventional client characteristics in an individualized form (IF condition: feedback individualized by a counselor; contact on demand) and a standardized form (SF condition: standardized feedback; contact on demand) of the same IBI for depression within a randomized controlled trial. To our knowledge, this is the first study exploring (a) if individualization of feedback leads to quantitatively and qualitatively different patterns of change and (b) if these change patterns show diverging associations with participants’ characteristics in a large clinical sample of adults provided with IBI for depression.

## 2 METHOD

### 2.1 Design and sample

Clients in this two‐arm assessor‐blind randomized controlled trial were recruited nationwide in Germany between March 2014 and March 2015 from the client base of a German public healthcare provider. The trial was prospectively registered (URL https://www.anzctr.org.au; ID: ACTRN12614000312640) and approved by the Research Ethics Committee of Freie Universität Berlin. Informed consent was obtained from all clients.

**Figure 1:**
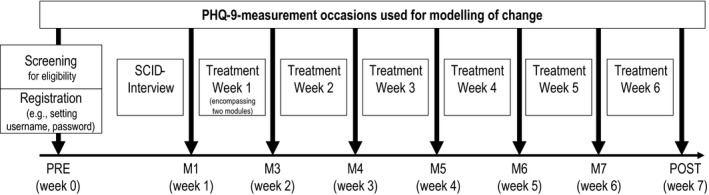
Study design with measurement occasions and associated time frame. Randomization occurred automatically after the completion of the SCID interview

Before the uptake of the first treatment module, participants had to complete a comprehensive online screening procedure (measurement occasion labeled “PRE”). Only nonsuicidal individuals with mild to moderate depression (Beck Depression Inventory‐II score between 14 and 28; score ≤1 on suicide item) were allowed to register for an account on the platform enabling them to make an appointment for a telephone‐administered structured clinical interview for DSM‐IV (SCID‐I, sections A through F; Wittchen, Zaudig, & Fydrich, 1997) within the next few days. Individuals with current mania, hypomania, or psychosis as assessed during this interview were excluded. After the completion of the interview, eligible participants were automatically randomized to one of the two treatment conditions. Within the next two days, they received a welcome message on the password‐protected platform and could start working with the intervention. Depressive symptom severity was assessed at the beginning of each week of treatment uptake. Due to the fact that treatment modules 1 and 2 were completed within the same week and all other modules took one week to complete, respective weekly measurement occasions are labeled as M1, M3, M4, M5, M6, and M7. Figure 1 provides an overview of all measurement occasions. A previous publication comparing the efficacy of the two treatment arms describes the recruitment strategy in more detail (Zagorscak, Heinrich, Sommer, Wagner, & Knaevelsrud, 2018). Overall, *N* = 1,089 individuals participated in the intervention. The mean age of the sample was 45.7 (*SD* = 11.3) years; 65.6% were female. A majority of individuals was married (51.5%), employed (88.2%), and highly educated (69.2% finished college‐preparatory school). Table 1 displays baseline sample characteristics. No significant differences between study conditions were found regarding any clinical or sociodemographic variables (all *p*‐values >.05).

**Table 1:** Sociodemographic and clinical sample characteristics at baseline

Variable		Total	IF	SF	*χ* ^2^	*p*
Sociodemographic characteristics						
Age^a^	*M* (*SD*)	45.7 (11.3)	45.7 (11.8)	45.8 (10.7)	0.166^b^	.868
Female sex	*n* (%)	714 (65.6)	360 (64.9)	354 (66.3)	0.246	.620
Unemployed	*n* (%)	127 (11.8)	59 (10.6)	68 (12.7)	1.169	.280
Lower level of formal education^c^	*n* (%)	336 (30.9)	160 (28.8	176 (33.3)	5.673	.140
Marital status						
Single	*n* (%)	373 (34.3)	196 (35.3)	177 (33.1)	1.301	.521
Married	*n* (%)	561 (51.5)	286 (51.5)	275 (51.5)		
Widowed/divorced	*n* (%)	155 (14.2)	73 (13.2)	82 (15.4)		
Clinical baseline characteristics						
PHQ‐9	*M* (*SD*)	11.8 (3.4)	11.9 (13.4)	11.7 (3.5)	1.044^b^	.297
SCID‐I diagnosis						
Current MDD	*n* (%)	458 (42.1)	247 (44.5)	211 (39.5)	3.194	.670
Remitted MDD	*n* (%)	285 (26.2)	137 (24.7)	148 (27.7)		
Dysthymia	*n* (%)	90 (8.3)	46 (8.3)	44 (8.2)		
Double depression	*n* (%)	58 (5.3)	27 (4.9)	31 (5.8)		
Bipolar or NOS^d^	*n* (%)	65 (6.0)	32 (5.8)	33 (6.2)		
No affective disorder Diagnosis (current/past)	*n* (%)	133 (12.2)	66 (11.9)	67 (12.5)		
Expectations^a^	*M* (*SD*)	10.0 (4.0)	9.9 (4.0)	10.1 (4.0)	0.462^b^	.644
Perseverative thinking^a^	*M* (*SD*)	37.4 (8.7)	37.2 (8.8)	37.6 (8.7)	0.739^b^	.460
Perceived social support	*M* (*SD*)	25.7 (5.0)	25.5 (5.0)	25.8 (5.0)	1.051^b^	.294

Note

*N* = 1,089.

Abbreviations: IF, individualized feedback (*n* = 555); NOS, not otherwise specified; PHQ‐9, Patient Health Questionnaire‐9; SCID‐I, structured clinical interview for DSM‐IV; SF, standardized feedback (*n* = 534).

a Variables have some missing values: age, *n* = 1,081; expectations, *n* = 1,081; PHQ‐9, *n* = 1,067.

b *t* test for independent samples.

c “lower” category encompasses no certificate or certificates from lower secondary/secondary school, “higher” category encompassing certificates from trade school/college‐preparatory school, college, or university.

d Individuals with bipolar disorders were included only if they were not experiencing current mania/hypomania.

### 2.2 Treatment

Clients were randomly assigned to one of two variants of an IBI for depression. Both conditions offered the same psychoeducation and intervention tools in seven modules (M1‐M7). In particular, clients completed two expressive writing tasks (M1‐M2, one week), behavioral activation through a daily planner (M3‐M4, two weeks), cognitive restructuring through thought protocols and interpretational bias training (M5‐M6, two weeks) as well as relapse prevention (M7, one week). Clients received either standardized feedback (*n*_SF_ = 534) or feedback individualized by a counselor (40 counselors, 21 holding a bachelor's degree and 19 holding a master's degree in psychology, *n*_IF_ = 555). Feedback was offered via written messages within a password‐protected Internet platform after the completion of each module. Clients in both intervention groups could receive contact on demand in case of technical problems or specific questions concerning the intervention.

### 2.3 Measures

*Categorical diagnoses of affective disorders* (e.g., current or past major depressive disorder (MDD), current dysthymia) were obtained from telephone‐administered structured clinical interviews (SCID‐I, sections A through F; Wittchen et al., 1997).

*Depressive symptom burden* was assessed with the nine‐item Patient Health Questionnaire‐9 (PHQ‐9; Kroenke, Spitzer, & Williams, 2001).

*The tendency for perseverative thinking* was measured using the 15‐item Perseverative Thinking Questionnaire (PTQ; Ehring et al., 2011).

*Expectations* were assessed with five seven‐point semantic differentials (Mendez, Rodrigues, Cornélio, Gallani, & Godin, 2010). The original item wording was slightly adapted to address expectations in the specific IBI context (e.g., “For me, participation in the IBI during the next six weeks would be “beneficial” to “harmful”).

*Perceived social support* was measured using the respective 8‐item subscale of the Berlin Social Support Scale (BSSS; Schulz & Schwarzer, 2003).

*Several sociodemographic characteristics* were assessed, that is, age and gender, level of education, employment, marital status, and history of psychotherapeutic treatment.

Clients completed the PHQ‐9 at baseline and after the completion of the intervention, as well as at the beginning of each week (at the beginning of M1, M3, M4, M5, M6, and M7). All other variables were assessed during baseline assessment only.

### 2.4 Statistical analysis

Overall, the analysis aimed to identify subgroups of clients with different patterns of change in depressive symptoms as measured with the PHQ‐9 in the IF and SF condition. The analysis had to consider that the two conditions might differ with regard to the number of change patterns and shape of the derived trajectories. GMM with latent base specifications was used for this purpose. The PHQ‐9 measurement structured the change process. A detailed description of the modeling process is available in the supporting online information (Appendix S1). In short, the modeling process comprised three steps: *First*, the optimal number of classes for each condition was determined separately using single‐group GMM. *Second*, to test for potential differences in change trajectories between conditions, multigroup GMM was used. *Third*, potential predictors of class membership, initial symptom load and interindividual differences in overall symptom change were included directly into the model (Asparouhov & Muthén, 2014). To avoid overburdening the model, we focused on a) baseline characteristics that are available in all studies (age, sex, education, and relationship status), and b) variables previously shown to influence depressive symptom change in psychological interventions and beyond (stress, social support, and expectations) (e.g., Brose, Wichers, & Kuppens, 2017; Constantino, Vîslă, Coyne, & Boswell, 2018; Gariépy, Honkaniemi, & Quesnel‐Vallée, 2016). In addition, instead of integrating another continuous measure of depression severity, we favored the inclusion of the SCID diagnosis as categorical measure of depression. Model selection was based on information criteria (AIC, aBIC, BIC, and CAIC). All models were estimated using M*Plus* 8.1 (Muthén & Muthén, 2017). Missing data were dealt with using FIML (depressive symptom load) and single‐value imputation (predictor variables).

## 3 RESULTS

### 3.1 Number of trajectory classes and patterns of change

#### 3.1.1 Single‐group GMM

The single‐group analyses pointed toward a two‐class solution in both intervention arms. Visual inspection indicated that the derived change patterns of both conditions showed considerable similarities. An illustration of the estimated change patterns of both classes together with estimated parameters separately for each intervention arm can be obtained from the supporting online information (Appendix S1: Figure S1, Table S1).

#### 3.1.2 Multigroup GMM

The multigroup analysis provided further evidence for the similarity of the derived classes across the two intervention conditions. All information criteria favored the more parsimonious model assuming no differences in change patterns between conditions (Appendix S1: Table S2). This result supports the notion that the intervention conditions do not differ regarding the number of classes, class sizes, and change trajectories. Therefore, class characterizations based on this constrained model are reported in the following.

*Class 1* (*delayed improvers*) comprises 37.5% of all randomized individuals. The average trajectory was marked by a low decrease in depressive symptoms (average initial symptom load: 12.4 points on the PHQ‐9; average pre‐ to postdecrease by 3.4 points, see Figure 1). The growth factor loadings of the first two measurement occasions were not significant (*λ* = −0.13, *p* = .298 and *λ* = 0.18, *p* = .220) indicating a rather stable average symptom load during early stages of the intervention. In other words, delayed improvers showed no early change in reference to overall improvement. The residual variances (i.e., scatter of the observed variables around the predicted curves) of delayed improvers were relatively large throughout the intervention ranging from 5.5 points (M6) to 9.1 points (post‐treatment). The numbers indicate that the observed individual trajectories are marked by ups and downs scattered around the individually predicted curve.

**Figure 2:**
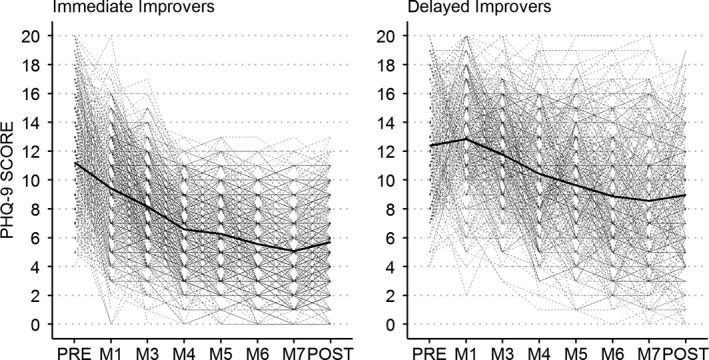
Observed patterns of change in the immediate improver class and the delayed improver class. Bold line depicts average change pattern

*Class 2* (*immediate improvers*) was the larger class and comprises 62.5% of the clients. The average symptom decrease in this class was larger than in class 1 (5.5 points on the PHQ‐9), while the average initial symptom load was similar (11.2 points). In contrast to class 1, immediate improvers went through a significant proportion of their average symptom improvement immediately after the initial screening. The growth factor loadings indicate that 33% of the average overall improvement had already occurred before intervention commenced (Slope‐loading at M1: *λ* = 0.33, *p* < .001). Immediate improvers showed the largest residual variances early during intervention ranging from 4.09 (M3) to 5.12 (pre‐assessment). The residual variances decreased toward the end of the intervention ranging from 1.44 (M6) to 3.02 (post‐treatment) indicating more stable symptom trajectories at this stage than in class 1. Table 2 summarizes estimated parameters for both classes, and the average change trajectories are illustrated in Figure 2.

**Table 2:** Model parameters of the single‐group and the constrained multigroup GMM Model

Parameter	Multigroup model	
	Delayed improvers	Immediate improvers
*λ*_k1_	0	0
*λ*_k2_	−0.13 (0.13)^NS^	0.33 (0.05)
*λ*_k3_	0.18 (0.15)^NS^	0.55 (0.04)
*λ*_k4_	0.57 (0.12)	0.84 (0.03)
*λ*_k5_	0.80 (0.07)	0.90 (0.02)
*λ*_k6_	1.03 (0.07)	1.02 (0.02)
*λ*_k7_	1.12 (0.05)	1.11 (0.02)
*λ*_k8_	1	1
*μ*_Ik_	12.39 (0.33)	11.23 (0.28)
*μ*_Sk_	−3.41 (0.76)	−5.54 (0.37)
*ψ*_Sk,Ik_	−2.23 (0.87)	−3.42 (1.04)
*ψ_Ik_*	5.75 (0.73)	6.27 (0.78)
*ψ_Sk_*	10.43 (2.48)	7.07 (1.15)
Var(*ε*_i1k_)	8.91 (1.60)	5.12 (0.96)
Var(ε_i2k_)	7.79 (1.94)	5.35 (1.33)
Var(*ε*_i3k_)	6.51 (1.46)	4.09 (0.87)
Var(*ε*_i4k_)	8.21 (1.14)	2.21 (0.30)
Var(*ε*_i5k_)	8.31 (1.71)	2.45 (0.32)
Var(ε_i6k_)	5.50 (1.17)	1.44 (0.30)
Var(ε_i7k_)	5.77 (1.08)	1.19 (0.22)
Var(ε_i8k_)	9.12 (1.56)	3.02 (0.40)

Note

*λ_kt_* = class‐ and time‐specific growth factor loading, where *k* = refers to the class and *t* to the measurement occasions. *μ*_Ik_ and *μ*_Sk_ = mean of the intercept and slope, respectively. *ψ_Ik_* and *ψ_Sk_* = variance of the intercept and slope. *ψ*_Sk,Ik_ = covariance between slope and intercept. Var(*ε*_itk_) = residual variance at the corresponding measurement occasion *t*. All parameters significant with *p* < .05 if not indicated otherwise. ^NS^ = nonsignificant.

### 3.2 Predictors of class membership and symptom course

Models were compared on the basis of information criteria. Results favored the use of a model that constrains the associations of predictors with initial symptom load and with the amount of symptom improvement to be equal across conditions and classes. For detailed results on model comparisons, see the supporting online information (Appendix S1, Table S2).

#### 3.2.1 Initial level of depressive symptom load across classes

When compared to individuals who did not receive any diagnosis in the SCID‐I, those who fulfilled the diagnostic criteria for MDD (SCID), *b* = 1.93, 95% CI [1.33, 2.52], *p* < .001, or double depression, *b* = 2.29, 95% CI [1.41, 3.18], *p* < .001, reported higher baseline depressive symptoms. Further, individuals with more severe perseverative thinking also reported higher baseline symptom severity, *b* = 0.07, 95% CI [0.05, 0.10], *p* < .001. In contrast, individuals with higher expectations toward the intervention showed lower initial depressive burden, *b* = −0.06, 95% CI [−0.11, −0.01], *p* = .012.

#### 3.2.2 Amount of overall symptom improvement across classes

On average, larger depressive symptom improvements over the course of the intervention were reported by individuals who fulfilled the criteria for MDD, *b* = −1.26, 95% CI [−1.97, −0.56], *p* < .001, and by individuals who reported more severe perseverative thinking *b* = −0.04, 95% CI [−0.06, −0.01], *p* = .008. In contrast, unemployed individuals experienced less improvement throughout the intervention, when compared to employed individuals, *b* = 0.67, 95% CI [0.01, 1.34], *p* = .048.

#### 3.2.3 Predictors of class membership

A current MDD diagnosis (SCID‐I), expectations, and perceived social support were statistically significant predictors of class membership. Individuals with higher initial expectations, OR = 1.09, 95% CI [1.01, 1.17], *p* = .020, and individuals with a current MDD diagnosis, OR = 2.68, 95% CI [1.34, 5.32], *p* = .005, showed increased odds of being classified as *delayed improvers*. Individuals with higher perceived social support showed increased odds of being classified as *immediate improvers*, OR = 1.08, 95% CI [1.04, 1.12],* p* < .001. All predictor variables and their associations with slope, intercept, and class membership are summarized in Table 3.

**Table 3:** Association between predictor variables and class‐specific intercepts, slopes, and class membership

Predictor	Intercept		Slope		Predict delayed response Class membership		
	*b* (*SE*)	*p*	*b* (*SE*)	*p*	*b* (*SE*)	*p*	OR [95% CI]
Age	0.01 (0.01)	.554	−0.01 (0.01)	.456	−0.02 (0.01)	.158	0.99 [0.96, 1.01]
Male sex	0.26 (0.20)	.181	−0.26 (0.24)	.275	0.14 (0.24)	.540	1.16 [0.73, 1.84]
Higher degree of formal education (Ref. lower)^a^	−0.10 (0.22)	.641	−0.21 (0.27)	.433	−0.27 (0.24)	.258	0.76 [0.47, 1.22]
Marital status (Ref.: single)							
Married	−0.21 (0.23)	.368	−0.29 (0.29)	.313	0.01 (0.27)	.963	1.01 [0.60, 1.71]
Widowed/divorced	0.29 (0.33)	.375	−0.64 (0.40)	.109	0.09 (0.34)	.786	1.10 [0.56, 2.14]
Prior psychotherapy (Ref.: no prior psychotherapy)	0.16 (0.19)	.412	−0.01 (0.23)	.970	0.39 (0.22)	.076	1.48 [0.96, 2.29]
Unemployed (Ref.: employed)	−0.39 (0.29)	.181	0.67 (0.34)	.048	−0.10 (0.33)	.764	0.90 [0.47, 1.74]
SCID‐I diagnosis (Ref.: no affective disorder)							
Current MDD	1.93 (0.30)	< .001	−1.26 (0.36)	<.001	0.98 (0.35)	.005	2.68 [1.34, 5.32]
Dysthymia	0.55 (0.39)	.152	0.32 (0.45)	.479	0.52 (0.46)	.262	1.67 [0.68, 4.12]
Remitted MDD	−0.16 (0.31)	.606	0.16 (0.36)	.663	0.49 (0.36)	.174	1.64 [0.81, 3.32]
Double depression	2.29 (0.45)	<.001	−0.28 (0.55)	.605	0.09 (0.55)	.873	1.09 [0.37, 3.17]
MDD NOS	0.53 (0.47)	.260	−0.08 (0.57)	.889	−0.11 (0.52)	.828	0.89 [0.32, 2.49]
Perseverative thinking	0.07 (0.01)	<.001	−0.04 (0.01)	.008	−0.00 (0.01)	.790	1.00 [0.97, 1.02]
Expectations	−0.06 (0.02)	.012	−0.03 (0.03)	.329	0.08 (0.04)	.020	1.09 [1.01, 1.17]
Perceived social support	−0.02 (0.02)	.346	−0.05 (0.03)	.069	−0.07 (0.02)	<.001	0.93 [0.89, 0.96]

Note

Beta‐regression weights as estimated in a model with class and group invariance. Ref., category used as reference; MDD, major depressive disorder; NOS, not otherwise specified.

a A“lower” category encompasses no certificate or certificates from lower secondary or secondary school and is contrasted against the “higher” category encompassing certificates from trade school/college‐preparatory school, college, or university.

## 4 DISCUSSION

The current study is the first to investigate and compare qualitatively and quantitatively discernable patterns of change in IBI for depression with varying levels of feedback individualization.

Across conditions, the study identified two groups of individuals that showed distinct average change patterns. Nearly two‐thirds of individuals randomized in the current trial belonged to an *immediate improver* class. Interestingly, this class size corresponds with response rates in previous studies on IBI for depression, which were summarized to range between 55% and 96% in a recent meta‐analysis (Königbauer, Letsch, Doebler, Ebert, & Baumeister, 2017). The depressive symptom change in this class is characterized by significant improvements after the initial screening phase with 33% of overall improvement taking place before the beginning of the first treatment module. On average, this class improved by 5.5 PHQ‐9 points overall, which is considered to be clinically significant change according to measure‐specific conventions (Titov et al., 2011).

In contrast, individuals in the second class were *delayed improvers* (37.5% of the sample). Depressive symptom change in this class is characterized by smaller symptom improvement (by 3.4 PHQ‐9 points) and by an initial treatment phase marked by stagnant symptom severity. These results complement the study by Lutz et al. (2017) in that they stress the importance of changes before and during early phases of IBI for depression and their association with overall outcome. Divergent from findings of the study at hand and two other studies on IBI for depression (Batterham et al., 2018; Sunderland et al., 2012), Lutz et al. (2017) found three discernable classes. There are some interesting similarities between the findings of the current study and the one published by Lutz et al. (2017). First, the average change pattern of the class Lutz et al. (2017) refers to as *early response after registration* (38.6%) matches well with our class of *delayed responders* (37.5%) with regard to the average symptom load at baseline (about 12 points) as well as with regard to average change across the first intervention modules. Lutz et al. (2017) report an average improvement of about 4 PHQ‐9 points within the first four weeks, which roughly corresponds to the 3.5 points observed in our sample. However, there are two major differences to the results reported in the current study. *First*, the immediate improvers’ class has a higher initial symptom load (about 11.2 points vs. about 8.5 points) than that reported for the *early response class* by Lutz et al. (2017). Consequently, our class of immediate improvers shows a steeper improvement from screening to M1 (1.8 points vs. about one point) within the period prior to starting the intervention. In addition, we did not find a class of individuals showing early deterioration. One explanation for these differences might be the different approaches to modeling class‐specific variation in change. While a latent base approach was used to represent change in the current study and the variance components were left unrestricted across classes, Lutz et al. (2017) used a log‐transformed scaling of time and added constraints on the variance of the intercept. Even though such constraints are often necessary to reach convergence, a simulation study has indicated that constraining variance components can lead to inflated numbers of classes (i.e., over‐extraction) (Diallo, Morin, & Lu, 2016). A simpler explanation might be that different samples taking part in different interventions might result in different class numbers. Considering that both studies found interesting differences between classes especially in the phases before the uptake of treatment began, further studies might address this aspect with more differentiated assessments.

Importantly, the results suggest that whether written feedback was individualized by a counselor or fully standardized did not influence the number of discernable subgroups or associated change patterns in otherwise identical intervention arms. These results are consistent with a recent meta‐analysis on the efficacy of IBI for individuals diagnosed with depression which did not find the presence of guidance to be a meaningful moderator of intervention success overall (Königbauer et al., 2017). Moreover, the current study extends the research by showing that not only the amount of pre‐ to postchanges is equal, but the average change patterns follow the same trajectories as well. Conversely, earlier meta‐analyses on pre‐ to postchanges during IBI for depression found feedback quantity and quality to be an essential contributor to treatment success (e.g., Johansson & Andersson, 2012; Richards & Richardson, 2012). Here, it is important to note that the study at hand investigated module‐wise change and change‐associated subgroups between two treatment conditions, which only differed in the degree feedback was individualized. Our study thus represents an encouragement to use GMM for the investigation of change patterns across more dissimilar forms of contact in IBI (e.g., guidance by telephone vs. standardized written guidance), which might result in divergent conclusions.

Regarding individuals’ characteristics associated with depressive symptoms and class membership, the results show that individuals who fulfill the criteria for MDD in a structured clinical interview show heightened baseline depressive symptom severity and larger improvement over time. That is not surprising, given that PHQ‐9 items are derived from the DSM‐IV criteria for depression (Kroenke et al., 2001). Furthermore, the finding is consistent with meta‐analyses on psychotherapy for depressive patients highlighting that the expected pre‐ to posteffect sizes (the amount of improvement) are lower for subclinical patients than those for individuals that fulfill the diagnostic criteria for MDD (Cuijpers, Karyotaki, et al., 2014; Cuijpers, Koole, et al., 2014). Interestingly, the presence of an MDD diagnosis is also associated with heightened odds of membership in the *delayed improver* class. In contrast to an individual that reports mild to moderate depressive symptoms on a questionnaire (PHQ‐9) only, an individual that further fulfills all criteria for a current MDD diagnosis might have a more complex symptom and comorbidity profile that decreases the probability of fast response to treatment (Melchior et al., 2016). While perseverative thinking was not associated with class membership, individuals with high levels of perseverative thinking reported more severe depressive symptoms at baseline and increased improvement. This finding is in line with several studies highlighting the importance of perseverative thinking for the prediction of symptoms of anxiety and depression (e.g., Spinhoven, van Hemert, & Penninx, 2018).

Regarding sociodemographic and psychosocial variables, the results demonstrate that unemployed individuals report lower symptom improvement than employed individuals, which is in accordance with previous results on the relationship between socioeconomic risk factors and depressive symptoms (e.g., Arias‐de la Torre, Vilagut, Martín, Molina, & Alonso, 2018). Moreover, individuals with higher perceived social support exhibit higher odds of being classified as an *immediate improver*. Apart from established cross‐sectional associations of social support and depression (e.g., Gariépy et al., 2016), previous studies demonstrated that individuals with low social support profit less from short‐term treatments and might benefit from treatment extension (Lindfors, Ojanen, Jääskeläinen, & Knekt, 2014). These findings stress that providers of IBI might increase response rates by identifying individuals with low social support and either improve their access to social resources or offer more extended treatment.

Finally, higher expectations were associated with lower baseline scores, a finding congruent with previous studies on baseline expectation symptom associations (e.g., Cohen, Beard, & Björgvinsson, 2015). These correlations may be explained through hopelessness that increases with depressive symptom severity (e.g., Horwitz, Berona, Czyz, Yeguez, & King, 2017) and dampens expectations for improvement (through treatment). Higher expectations were further associated with membership in the delayed improver class. Given that the contents and procedures of IBIs are still mostly unknown to the public (Apolinário‐Hagen, Vehreschild, & Alkoudmani, 2017), some clients may have unrealistic expectations toward treatment. As a consequence, initial disappointment might reduce the probability of experiencing rapid improvement (Greer, 1980). Overall, these findings stress the importance of assessing expectations in IBI for depression in order to react to expectations that might be either unrealistic or pessimistic. While a recent study highlighted that expectations might change during treatment (Vîslă, Flückiger, Constantino, Krieger, & Holtforth, 2018), there are no studies on how expectations develop through the course of IBI for depression. Thus, future studies should assess expectations at multiple time points to further explore the expectation symptom course interplay.

Some other directions for future research can be derived from the limitations of the present study design. The findings of this study pertain to cognitive‐behavioral IBI that utilizes written feedback (standardized vs. individualized) and includes mildly to moderately depressed individuals only. Patterns of change and associated individual characteristics might differ in other populations, in forms of treatment that apply other qualities or quantities of feedback or use treatment techniques that might entail other change trajectories (e.g., interpersonal or psychodynamic treatments). Furthermore, this study is limited to exploring change patterns derived using PHQ‐9 sum scores. While this is standard in clinical research and practice, it might cover up relevant changes on the symptom level (i.e., cognitive symptom change during modules targeting cognitive restructuring). Thus, a fruitful direction for future studies would be more symptom‐oriented modeling of depression and depression change (e.g., Heinrich, Zagorscak, Eid, & Knaevelsrud, 2018).

## 5 CONCLUSION

Individualizing feedback did not influence patterns of change when compared to standardized feedback, and a majority of clients showed immediate improvements in both treatment conditions. However, a smaller group was at risk of delayed and reduced improvements. Fruitful directions for clinicians aiming to increase improvements during IBI are expectation management, treatment extension, and a bolstering of socially supportive relationships.

## CONFLICT OF INTEREST

The authors do not report any conflicts of interest related to this publication.

## Data Availability

Data are available on request due to privacy/ethical restrictions.
