# Supplementary material for: How individuals change during internet‐based interventions for depression: A randomized controlled trial comparing standardized and individualized feedback
Source: Brain Behav. 2019 Nov 27;10(1):e01484. doi: 10.1002/brb3.1484 (PMC6955845; doi:10.1002/brb3.1484)
Supplement: Supplementary file 1 [file BRB3-10-e01484-s001.docx]

**Supporting Online Material – Appendix S1.**

In the first section of the supporting information we present a detailed description of the applied statistical methods and the steps taken during the analysis. The second section of the supplement offers a more detailed explanation and extension of the results of the single-group, the multi-group and the predictor analysis.

**Statistical Methods**

***Analysis.*** We used Growth Mixture Models (GMM; Muthén & Shedden, 1999; Ram & Grimm, 2009; please see Morin for an introduction to GMM) with latent base-specification (first and last slope-factor loading fixed at 0 and 1, respectively) to investigate whether the data set contains subgroups of individuals characterized by similar average change trajectories in depressive symptom load (Morin et al., 2011; Morin, Maiano, Marsh, Nagengast, & Janosz, 2013). The latent base specification allows for the modeling of non-linear change (Morin et al., 2011). We used PHQ-9 measurements obtained at the beginning of each specific treatment module to structure the change process. Importantly, the current data set contains data from two separate treatment conditions. Each treatment condition might lead to different patterns of symptom changes in participating patients. Consequently, the number of classes and the quantitative features of these classes (e.g., different amount of change) might differ across conditions. Therefore, a modeling approach considering these conditions seemed necessary (Muthén, Brown, Leuchter, & Hunter, 2008).

*In a first step*, we analyzed each treatment condition separately to account for the possibility that both treatment conditions show a different number of classes (single-group GMM). As suggested by Diallo, Morin, and Lu (2016), we used a rather unrestricted GMM for class-enumeration. Means (*μ_Sk_* and *μ_Ik_*; *I* represents the intercept-factor and *S* represents the slope-factor), variances (*_Ik_* and *_Sk_*), and co-variances (*_Ik,Sk_*)), were estimated class-specific (*k =* 1, …, *K, K =* number of classes), while slope-loadings (λ*_tk_*; *t* = *1*, … , *T*; *T* = number of measurement occasions) and the residual terms of the indicators were estimated class and time-specific (*ε_itk_*). Only if estimation difficulties occurred (best-likelihood not replicated, negative variances, excessively small classes) and the information criteria did not favor a specific model, more restrictive models were tested. These models included constraints on the residual terms (ε*_ik_*, ε*_it_* or ε*_i_*). Recent simulation studies have shown, that unrestricted models are beneficial in identifying the appropriate number of classes and help to avoid small classes which might represent artefacts of unnecessary restrictions on variance or co-variance parameters of the model (Diallo et al., 2016, 2017; Peugh & Fan, 2012). Following the suggestions by several groups of authors, the optimal number of classes for each treatment condition was determined on the basis of several statistical information criteria (*Akaike Information Criteria*, AIC; *Corrected AIC*, CAIC; *Bayes Information Criteria*, BIC; *sample-size adjusted* BIC, aBIC; *Vuo-Long-Mendel Likelihood Ratio-Test*, VLM-LRT), the appropriateness of the estimated model parameters as well as the interpretability, distinctiveness and sizes of derived classes to decide for the optimal number of classes (Masyn, 2013; Meyer & Morin, 2016; Morin et al., 2011). In line with recommendations from recent studies, we decided to do class enumeration using an unconditional GMM, that is without inclusion of covariates (Diallo et al., 2017; Masyn, 2013).

*In a second step*, we specified a multi-group GMM taking the known membership of individuals to two distinct treatment conditions and the number of change patterns derived in the single group-analysis into account (*g =* SF or *g* = IF). The multi-group approach allows for the testing of potential differences in the configuration of change patterns and class sizes across treatment conditions. In other words, the MG approach provides answers to the question as to whether the provision of qualitatively different feedback (i.e., individualized vs. standardized) leads to differences in average change of classes. We used a model with the optimal number of classes for each treatment condition as determined by the single-class analysis with group and class-specific means (*μ_Skg_* and *μ_Ikg_*), variances (*_Ikg_* and *_Skg_*), co-variances (*_Ikg,Skg_*)) as baseline-model. Furthermore, slope loadings (*λ_tkg_*) and residuals (*ε_itkg_*) were estimated separately and specifically for each treatment condition, class and measurement occasion. Class sizes were allowed to vary across treatment conditions. We compared this model against a model assuming the same configuration of means, variances, co-variances and residuals for corresponding classes across treatment conditions with equal class sizes across treatment conditions (*μ_Sk_*, *μ_Ig_* *_Ik_*, *_Sk_*, *_Ik,Sk_, λ_tk_, ε_itk_*). We used information criteria to decide on whether the unrestricted or restricted model should be favored.

*In a third step*, predictor variables for slope and intercept, as well as class-membership were included directly into the model. Therefore, the models consider inaccuracy of class-assignment properly. In a baseline model, all regression weights were allowed to vary group- and class-specific (slope regression-weights: *β_xSkg_*_;_ intercept regression-weights: *β_xIkg_*) which is comparable to including an interaction term into multiple regression. A more restricted model assuming equal regression weights across classes and treatment conditions (*β*xI and *β*xS) followed this baseline model. We evaluated information criteria to decide which model should be favored. Given the exploratory fashion of the analysis, we favored parsimony when comparing models.

All models were estimated with M*plus* 8.1 (Muthén & Muthén, 1998-2017; normal distribution, 10’000 initial starts, 500 stage optimizations and 500 initial stage iterations). Missing data on the PHQ-9 scores were dealt with using the full information maximum likelihood estimation procedure, while missing values on predictor variables were replaced using a single value imputation.

**Results of Single-Group GMM**

***Individualized Feedback-Condition.*** Models with 1 to 3 classes with class-specific, means (*μ_Sk_* and *μ_Ik_*), slope (*_Sk_*) and intercept variances (*_Ik_*), co-variances (*_SkIk_*) as well as class- and time-specific factor loadings (*λ_tk_*) and residuals (*ε_itk_*) converged properly. The best likelihood replicated several times. All parameter estimates were within a plausible range (no variances smaller than zero, no inflated standard errors). However, the best likelihood value of the 4-class solution could not be replicated even after increasing the number of random starts. Since BIC and CAIC reached a plateau at the 3-class solution, no further models with additional restrictions models were estimated and class enumeration was performed considering the statistically sound models with one to three classes (all information criteria are summarized in Table S2). The AIC and sBIC were uninformative and decreased continuously from the 1-class to the 3-class-soluation. While the VLM-LRT favored a 3-class solution over a 2-class-soluation (still significant after adding a third class), the BIC and CAIC favored a 2-class-solution. Visual inspection of the derived change patterns showed large similarities of two classes in the 3-class solution. Therefore, we favored the 2-class model as the most parsimonious one.

Class 1 (*delayed improvers*) comprises 46.0% of the participants. The average trajectory is marked by an average improvement of *μ_S1_* = -3.5, which is below the cut-off suggested for reliable change using the PHQ-9 (Titov et al., 2011). Class 2 (*immediate improvers*) comprises 54.0% of the participants. The average symptom improvement in this class was *μ_S2_* = -6.2 points. Interestingly, these two classes differ in their early symptom development. *Immediate improvers* showed a significant proportion of their average symptom improvement immediately after the SCID-I interview and prior to treatment uptake at Ml (*λ_12_* = 0.266, *p* < .001), while no such changes occurred in the class of delayed improvers (λ*_11_* = 0.071, *p* = .626). Both classes showed not only considerable heterogeneity in initial symptom-load (*_I1_* = 6.1, *_I2_* = 7.9) but in changes throughout the intervention (*_S1_* = 9.3, *_S2_* = 9.4). Supp. Table S1 summarizes all estimated parameters. Supp. Figure S1 (Panel IF-condition) visualizes the derived change patterns.

***Standardized Feedback-Condition.*** The 1- and 2-class solution with class-specific means (*μ_Sk_* and *μ_Ik_*), slope (*_Sk_*) and intercept variances (*_Ik_*), co-variances (**_SkIk_) as well as class- and time-specific factor loadings (λ*_tk_*) and residuals (ε*_itk_*) converged properly. The best likelihood replicated several times and all parameter estimates were in a plausible range. The best likelihood value of the 3-class solution did not replicate. In order to determine whether a more parsimonious 3-class model fits the data better, additional restrictions were added. A model with class-specific but time-unspecific residuals did not converge and the best likelihood value could not be replicated (ε*_ik_*). However, models with error variances constrained to be equal across classes and measurement occasions (ε*_i_*) as well as across classes but specific for each measurement occasion (ε*_it_*) converged and were considered during the model selection process. The statistical information criteria (AIC, BIC, SA-BIC, CAIC, see Supp. Table S2) pointed to the more complex 2-class model. Therefore, we selected this model as optimal solution for the SF group (see Figure S1).

Class 2 (*immediate improvers*) comprises 64.2% of the participants. The average improvement in the class was *μ_S2_* = -5.5 points on the PHQ-9, which is descriptively larger than the 5 points reliable change benchmark of the PHQ-9 (Titov et al., 2011). Class 1 (*delayed improvers*) comprises 35.8% of the participants. The average improvement throughout the interventions was smaller (*μ_S1_* = -2.4) than in class one. As in the IF condition, the classes showed considerable difference in the symptom course at early treatment periods. Individuals in the *immediate improvers* class started to show a significant proportion of their average symptom improvement immediately after the SCID-I interview and prior to starting to treatment uptake at Ml (λ*_12_* = 0.33, *p* < .001) while no beneficial changes occurred in class 1 (λ*_11_* = -0.48, *p* < .001; *_21_* = -0.33, *p* < .001). In contrast, the trajectory showed a slight increase in symptom load followed by improvements later throughout the intervention. Again, both classes showed not only significant heterogeneity in initial symptom-load (*_I1_* = 6.3, *_I2_* = 6.2) but in changes throughout the intervention (*_S1_* = 7.2, *_S2_* = 5.6). Supp. Table S1 summarizes all estimated parameters. Supp. Figure S1 (Panel SF-Condition) visualizes the change patterns.

**Results of Multi-Group GMM**

The single group analysis pointed towards two classes in each treatment condition. Based on visual inspection, the two classes of both groups showed considerable similarities. *First*, both treatment conditions comprised a class of individuals showing constant improvement throughout the whole treatment. *Second,* we observed a class of individuals with delayed and lower overall-improvements in both treatment groups. Based on these results, we specified a two class-model for each treatment condition. *In a first step*, we allowed class-sizes, (*μ_Skg_* and *μ_Ikg_*), slope (*_Skg_*) intercept variances (*_Ikg_*) and co-variances (_Sk_*_g,_*_Ik_*_g_*) to vary across groups and classes. Additionally, factor loadings (λ*_tkg_*) and residuals (ε*_itkg_*) were allowed to vary across treatment conditions, classes and time-points. The model converged and the best likelihood values were replicated several times. To check for differences between the treatment conditions, we estimated a more constrained model assuming that not only the numbers of classes are equal, but the configuration of class-specific average change patterns and their sizes were constrained to be equal across the different treatment conditions as well (e.g. there is no condition-specificity, *μ_Sk_*, *μ_Ik_* *_Ik_*, *_Sk_*, *_Ik,Sk_, λ_tk_, ε_itk_*). All information criteria pointed towards the more parsimonious model assuming no condition-specificity. This suggests that the patterns of change between both treatment conditions are highly similar. Figure 3 depicts the derived trajectories for restricted and unrestricted versions of the model.

Results of Multi-Group Modeling, including descriptions of the two classes are provided in the manuscript.

**Results of the Predictor Analysis**

First, we estimated an unconstrained model estimating the classes within each treatment condition freely (*λ_tkg_, _Skg_, _Ikg_, _Skg,Ikg,_ ε_itkg_*). Predictors of slopes (*β_xSg_*) and intercepts (*β_xIg_*) were allowed to vary across treatment conditions, while they were restricted to be equal across condition-specific classes. A model constraining shape of change trajectories and regression weights of predictor variables to be equal across classes and treatment conditions (*β_xg_* and *β_xg_*_,_ respectively) was estimated in a second step. Again, both models resulted in proper parameter estimates and the best likelihood value was replicated several times. The shape of the derived trajectories did not change. All information criteria clearly favored the most parsimonious model with equal regressive relations across classes and treatment conditions. Therefore, it seems reasonable to assume, that symptom change in the two treatment arms does not only follow the same trajectories but also shares the same predictors. We present a detailed discussion of the estimated paths in the manuscript.

In order to illustrate and contextualize the meaning of the OR in this setting, we would like to provide a comparative example: Current MDD is a dichotomous variable, but expectations are measured with sums of longer scales. Fulfilling the criteria for a current MDD increases the odds of being in the delayed responder class by a factor of 2.8 (OR_MDD_ = 2.8). In order to achieve such an increase in the odds, the individually reported expectations need to be raised by 12 units of measurement (OR_EXP_ = 1.09; 1.09^12^ = 2.81).

| *Table S1.* Model Parameters of the Single-Group GMM and the constrained Multi-Group GMM models. | | | | | | | | |
| --- | --- | --- | --- | --- | --- | --- | --- | --- |
|  | IF-Condition | |  | SF-Condition | |  | MG-Model | |
| Parameter | Delayed Improvers | Immediate Improvers |  | Delayed Improvers | Immediate Improvers |  | Delayed Improvers | Immediate Improvers |
|  | Est (SE) | Est (SE) |  | Est (SE) | Est (SE) |  | Est (SE) | Est (SE) |
| *λ*_k1_ | 0 | 0 |  | 0 | 0 |  | 0 | 0 |
| *λ*_k2_ | 0.07 (0.15)^NS^ | 0.27 (0.05) |  | -0.48 (0.15) | 0.33 (0.07) |  | -0.13 (0.13) ^NS^ | 0.33 (0.05) |
| *λ*_k3_ | 0.34 (0.14) | 0.54 (0.04) |  | -0.32 (0.15) | 0.57 (0.07) |  | 0.18 (0.15) ^NS^ | 0.55 (0.04) |
| *λ*_k4_ | 0.60 (0.14) | 0.83 (0.03) |  | 0.34 (0.16) | 0.86 (0.04) |  | 0.57 (0.12) | 0.84 (0.03) |
| *λ*_k5_ | 0.78 (0.11) | 0.92 (0.03) |  | 0.78 (0.13) | 0.87 (0.03) |  | 0.80 (0.07) | 0.90 (0.02) |
| *λ*_k6_ | 1.01 (0.09) | 1.02 (0.02) |  | 0.98 (0.12) | 1.06 (0.04) |  | 1.03 (0.07) | 1.02 (0.02) |
| *λ*_k7_ | 1.08 (0.07) | 1.09 (0.02) |  | 1.13 (0.09) | 1.17 (0.03) |  | 1.12 (0.05) | 1.11 (0.02) |
| *λ*_k8_ | 1 | 1 |  | 1 | 1 |  | 1 | 1 |
| *μ*_Ik_ | 12.35 (0.27) | 11.37 (0.28) |  | 11.78 (0.31) | 11.41 (0.21) |  | 12.39 (0.33) | 11.23 (0.28) |
| *μ*_Sk_ | -3.46 (0.49) | -6.23 (0.45) |  | -2.37 (0.54) | -5.45 (0.32) |  | -3.41 (0.76) | -5.54 (0.37) |
| **_Sk,Ik_ | -2.50 (0.95) | -5.80 (1.30) |  | -0.74 (0.81) | -2.99 (0.90) |  | -2.23 (0.87) | -3.42 (1.04) |
| *_Ik_* | 6.06 (0.88) | 7.87 (1.17) |  | 6.27 (1.03) | 6.15 (0.86) |  | 5.75 (0.73) | 6.27 (0.78) |
| *_Sk_* | 9.30 (1.91) | 9.43 (1.36) |  | 7.24 (1.95) | 5.64 (1.04) |  | 10.43 (2.48) | 7.07 (1.15) |
| Var(*ε*_i1k_) | 6.40 (1.26) | 4.51 (0.99) |  | 8.61 (1.72) | 6.21 (1.07) |  | 8.91 (1.60) | 5.12 (0.96) |
| Var(*ε*_i2k_) | 7.47 (1.45) | 6.46 (1.23) |  | 3.65 (1.20) | 7.79 (1.41) |  | 7.79 (1.94) | 5.35 (1.33) |
| Var(*ε*_i3k_) | 5.33 (0.76) | 4.45 (0.66) |  | 4.74 (1.16) | 4.43 (1.18) |  | 6.51 (1.46) | 4.09 (0.87) |
| Var(*ε*_i4k_) | 7.28 (1.33) | 1.99 (0.34) |  | 8.35 (1.40) | 2.40 (0.34) |  | 8.21 (1.14) | 2.21 (0.30) |
| Var(*ε*_i5k_) | 6.92 (1.43) | 1.86 (0.42) |  | 9.01 (3.59) | 3.53 (0.75) |  | 8.31 (1.71) | 2.45 (0.32) |
| Var(*ε*_i6k_) | 4.72 (0.95) | 0.84 (0.35) |  | 6.88 (2.24) | 1.91 (0.33) |  | 5.50 (1.17) | 1.44 (0.30) |
| Var(*ε*_i7k_) | 6.13 (1.26) | 1.03 (0.41) |  | 5.11 (1.27) | 1.12 (0.31) |  | 5.77 (1.08) | 1.19 (0.22) |
| Var(*ε*_i8k_) | 8.88 (1.44) | 2.25 (0.79) |  | 8.44 (2.50) | 3.85 (0.63) |  | 9.12 (1.56) | 3.02 (0.40) |
| *Note. λ_kt_* = class and time specific growth factor-loading, where *k* = refers to the class and *t* to the measurement occasions. *μ*_Ik_ and *μ*_Sk_ = mean of the intercept and slope, respectively. *_Ik_* and *_Sk_* = variance of the intercept and slope. **_Sk,Ik_ = covariance between slope and intercept. Var(*ε*_itk_) = residual variance at the corresponding measurement occasion *t.* All parameters significant with *p* < .05 if not indicated otherwise.  ^NS^ = non-significant. | | | | | | | | |

| *Table S2.* Information Criteria and Entropy for Each of the Estimated Models. | | | | | | | | | |
| --- | --- | --- | --- | --- | --- | --- | --- | --- | --- |
| Model | #par | logL | scaling | AIC | BIC | aBIC | CAIC | VLM-LRT | entropy |
| IF-Group ^a^ |  |  |  |  |  |  |  |  |  |
| 1 class | 19 | -9584.172 | 1.294 | 19206 | 19288 | 19228 | 19320 | --- | --- |
| 2 classes | 39 | -9417.485 | 1.369 | 18913 | 19081 | 18958 | 19147 | .014 | 0.556 |
| 3 classes | 59 | -9356.155 | 1.195 | 18830 | 19085 | 18898 | 19184 | .013 | 0.547 |
| SF-Group |  |  |  |  |  |  |  |  |  |
| 1 class | 19 | -9013.258 | 1.590 | 18065 | 18146 | 18086 | 18178 | --- | --- |
| 2 classes | 39 | -8861.870 | 1.425 | 17802 | 17969 | 17845 | 18035 | .011 | 0.571 |
| 3 classes ^b^ | 36 | -8915.305 | 1.275 | 17903 | 18057 | 17942 | 18118 | .186 | 0.487 |
| 3 classes ^c^ | 43 | -8888.899 | 1.422 | 17864 | 18048 | 17911 | 18121 | .538 | 0.650 |
| MG-Group |  |  |  |  |  |  |  |  |  |
| 2 classes | 79 | -19033.990 | 1.392 | 38226 | 38620 | 38370 | 38699 | --- | 0.782 |
| 2 classes, constrained | 40 | -19068.615 | 1.463 | 38217 | 38417 | 38290 | 38457 | --- | 0.769 |
| MG-Group incl. Predictors |  |  |  |  |  |  |  |  |  |
| 2 classes | 85 | -18912.895 | 1.221 | 37996 | 38420 | 38150 | 38505 | --- | 0.787 |
| 2 classes, constrained | 213 | -18811.701 | 1.191 | 38049 | 39113 | 38436 | 39326 | --- | 0.823 |
| *Note.*  ^a^ Models were estimated with class specific means, variances, covariance’s and class- and time-specific loadings and variances if not stated otherwise.  ^b^ additional constraint: ε*_i_* ^c^  additional constraints added: ε*_it_* | | | | | | | | | |


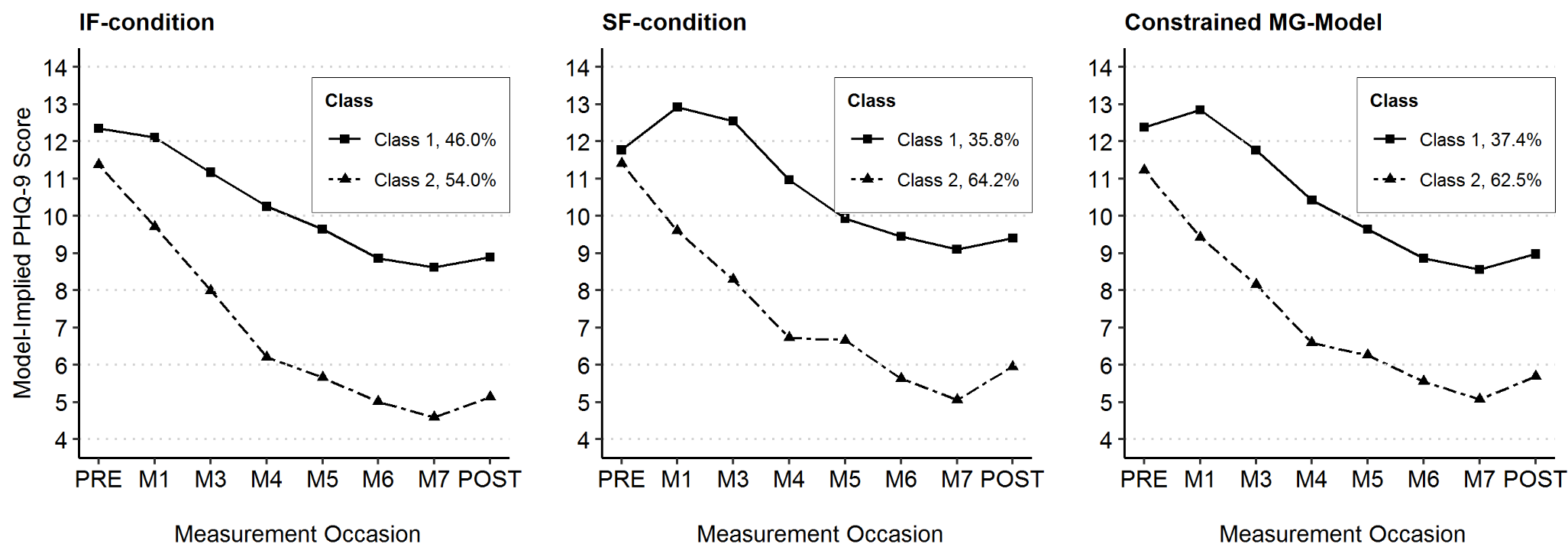


*Figure S1.* Estimated Change Pattern of the Single-Group Analysis (IF-Condition and SF) Condition and the Constrained Multi-Group Model

**References**

Diallo, T. M. O., Morin, A. J. S., & Lu, H. (2016). Impact of Misspecifications of the Latent Variance–Covariance and Residual Matrices on the Class Enumeration Accuracy of Growth Mixture Models. *Structural Equation Modeling: a Multidisciplinary Journal*, *23*, 507–531. https://doi.org/10.1080/10705511.2016.1169188

Diallo, T. M. O., Morin, A. J. S., & Lu, H. (2017). The impact of total and partial inclusion or exclusion of active and inactive time invariant covariates in growth mixture models. *Psychological Methods*, *22*, 166–190. https://doi.org/10.1037/met0000084

Masyn, K. E. (2013). Latent Class Analysis and Finite Mixture Modeling. *The Oxford Handbook of Quantitative Methods*, 551.

Meyer, J. P., & Morin, A. J.S. (2016). A person-centered approach to commitment research: Theory, research, and methodology. *Journal of Organizational Behavior*, *37*, 584–612. https://doi.org/10.1002/job.2085

Morin, A. J. S., Maiano, C., Marsh, H. W., Nagengast, B., & Janosz, M. (2013). School life and adolescents' self-esteem trajectories. *Child Development*, *84*, 1967–1988. https://doi.org/10.1111/cdev.12089

Morin, A. J. S., Maïano, C., Nagengast, B., Marsh, H. W., Morizot, J., & Janosz, M. (2011). General Growth Mixture Analysis of Adolescents' Developmental Trajectories of Anxiety: The Impact of Untested Invariance Assumptions on Substantive Interpretations. *Structural Equation Modeling: a Multidisciplinary Journal*, *18*, 613–648. https://doi.org/10.1080/10705511.2011.607714

Muthén, B., & Shedden, K. (1999). Finite mixture modeling with mixture outcomes using the EM algorithm. *Biometrics*, *55*, 463–469.

Muthén, B., Brown, H., Leuchter, A., & Hunter, A. (2008). General approaches to analysis of course: applying growth mixture modeling to randomized trials of depression medication. *Causality and Psychopathology: Finding the Determinants of Disorders and Their Cures. Washington, DC: American Psychiatric Publishing*, 159–178.

Muthén, L. K., & Muthén, B. (1998-2017). *Mplus User’s Guide.: Eighth Edition.* Los Angeles, CA: Muthén & Muthén.

Peugh, J., & Fan, X. (2012). How Well Does Growth Mixture Modeling Identify Heterogeneous Growth Trajectories? A Simulation Study Examining GMM's Performance Characteristics. *Structural Equation Modeling: a Multidisciplinary Journal*, *19*, 204–226. https://doi.org/10.1080/10705511.2012.659618

Ram, N., & Grimm, K. J. (2009). Growth Mixture Modeling: A Method for Identifying Differences in Longitudinal Change Among Unobserved Groups. *International Journal of Behavioral Development*, *33*, 565–576. https://doi.org/10.1177/0165025409343765

Titov, N., Dear, B. F., McMillan, D., Anderson, T., Zou, J., & Sunderland, M. (2011). Psychometric comparison of the PHQ-9 and BDI-II for measuring response during treatment of depression. *Cognitive Behaviour Therapy*, *40*, 126–136. https://doi.org/10.1080/16506073.2010.550059
